# Report on Medical Literature

**Published:** 1873-03-15

**Authors:** 


REPORT ON MEDICAL LITERATURE.—Authors and publishers who have issued new books, or new editions of old ones, during the past year, are requested to furnish copies of the same to the chairman of the committee on medical literature, appointed at the last meeting of the American Medical Association, Prof. Austin Flint, of New York city.

---

CASE OF GASTROTOMY.—Dr. Troup (*Edin. Med. Journal*, July, 1872) describes a case in which gastrotomy was resorted to in a case of impermeable stricture of the œsophagus. He was assisted by Dr. David Lyell, of Newburgh, and Dr. John Lyell, of Glasgow. The patient was at the point of starvation, and suffering from intense thirst, and begged to have the operation performed. A vertical incision was made to the left of the median line, over the region of the stomach, and a tracheotomy tube inserted, the margin of the opening in the stomach being stitched to the parietes of the abdomen. Milk and stimulants were by this means readily passed into the stomach, and the three remaining days of the man's life were spent in comparative comfort. A *post mortem* examination revealed the presence of a large epitheliomatous mass, at the cardiac end of the stomach.

